# Assessing effectiveness of many-objective evolutionary algorithms for selection of tag SNPs

**DOI:** 10.1371/journal.pone.0278560

**Published:** 2022-12-08

**Authors:** Rashad Moqa, Irfan Younas, Maryam Bashir

**Affiliations:** FAST School of Computing, National University of Computer and Emerging Sciences, Lahore, Pakistan; Torrens University Australia, AUSTRALIA

## Abstract

**Background:**

Studies on genome-wide associations help to determine the cause of many genetic diseases. Genome-wide associations typically focus on associations between single-nucleotide polymorphisms (SNPs). Genotyping every SNP in a chromosomal region for identifying genetic variation is computationally very expensive. A representative subset of SNPs, called tag SNPs, can be used to identify genetic variation. Small tag SNPs save the computation time of genotyping platform, however, there could be missing data or genotyping errors in small tag SNPs. This study aims to solve Tag SNPs selection problem using many-objective evolutionary algorithms.

**Methods:**

Tag SNPs selection can be viewed as an optimization problem with some trade-offs between objectives, e.g. minimizing the number of tag SNPs and maximizing tolerance for missing data. In this study, the tag SNPs selection problem is formulated as a many-objective problem. Nondominated Sorting based Genetic Algorithm (NSGA-III), and Multi-Objective Evolutionary Algorithm based on Decomposition (MOEA/D), which are Many-Objective evolutionary algorithms, have been applied and investigated for optimal tag SNPs selection. This study also investigates different initialization methods like greedy and random initialization. optimization.

**Results:**

The evaluation measures used for comparing results for different algorithms are Hypervolume, Range, SumMin, MinSum, Tolerance rate, and Average Hamming distance. Overall MOEA/D algorithm gives superior results as compared to other algorithms in most cases. NSGA-III outperforms NSGA-II and other compared algorithms on maximum tolerance rate, and SPEA2 outperforms all algorithms on average hamming distance.

**Conclusion:**

Experimental results show that the performance of our proposed many-objective algorithms is much superior as compared to the results of existing methods. The outcomes show the advantages of greedy initialization over random initialization using NSGA-III, SPEA2, and MOEA/D to solve the tag SNPs selection as many-objective optimization problem.

## 1 Introduction

Living organisms have Deoxyribonucleic acid (DNA) which carries genetic instructions and all genetic recipes of the living organisms. The DNA has two strands called Polynucleotides; which are built using simple units called nucleotides [1]. A single nucleotide can be one of four types namely, Adenine (A), Cytosine (C), Guanine (G), or Thymine (T). The double-stranded DNAs are bounded together using the nitrogenous bases, according to the base-pairing rules (A with T, and C with G) [1]. Human DNA is organized into packaged units called chromosomes (46 single or 23 pairs of chromosomes in humans). Due to the complexity and a large number of DNA sequences, scientists face difficulties in the study of genetic instructions, so scientists have mapped the entire DNA to a single sequence called Genome which has all the genetic material of a given organism [1]. The stored human genome is just like a book, where the book (genome) contains 23 chapters (chromosomes), and each chapter would have 48 to 250 million letters (A, T, C, G) without spaces [1]. The entire genome book will have over 3.2 billion letters. Most cells of the human body will have at least one copy of the book (genome). The genome sequence of any two humans has 99.9% similarity [1]. The 0.01% variation between genome sequences of two individuals is what makes one individual different than another. At a specific base position in the human genome, most individuals may have base A, but few individuals may have base T [1]. The variation at the base position of the genome is called a single-nucleotide polymorphism (SNP), and the two possible nucleotide variations (A or T) are the alleles of that base position [2]. Each SNP can have four alleles (A, C, G, T), but in most cases, only two possible alleles occur in a single SNP [2].

In genetics, a genome-wide association study is an observational study of a genome-wide set of genetic variants in different individuals. Genetic variants of humans can affect how humans develop diseases and respond to drugs and vaccines. The genome-wide association typically focuses on associations between SNPs and traits like major human diseases, but can equally be applied to any other organism. The Human Genome Project (an international scientific research project to determine the sequence of nucleotide base pairs that make up human DNA), found that there are 1.42 million SNPs [3] in the human genome. It is very expensive and time-consuming for genotyping platforms to study every individual SNP (for example brca2 gene has around 10 thousand SNPs). It is possible to identify genetic variation and association to phenotypes without genotyping every SNP [4, 5] in a chromosomal region, so it is important to find a subset of SNPs that can represent all SNPs, this subset is called tag SNPs. Tag SNPs represent the original SNPs in a region of the genome. It is important to select tag SNPs that are few and are the best representatives. Currently, most tag SNPs selection algorithms use a block-based approach, which is based on linkage disequilibrium (LD) among all SNPs [4, 5]. Linkage disequilibrium is the non-random association of alleles at different loci in a given population [4]. The human genome is composed of high-LD blocks called haplotype blocks [2]. Although, small tag SNPs save the computation time of genotyping platform, there could be missing data or genotyping errors in small tag SNPs [6, 7]. The set of tag SNPs should not be very small to reduce the error rate. Moreover, the distances between the different haplotype patterns are also taken into consideration to balance selection among all patterns. Hence, tag SNPs selection is a multi-objective problem with some trade-offs between objectives, e.g. minimizing the number of tag SNPs and maximizing tolerance for missing data.

The problem of finding minimum robust tag SNPs is shown to be NP-hard [6]. To find robust tag SNPs efficiently, many algorithms have been proposed, and among these methods, evolutionary algorithms have shown promising results. Previous approaches have mostly used multi-objective algorithms for solving this problem [8, 9]. For Two-Three objectives, multi-objective algorithms are reasonable enough but as the number of objectives increases to four or more, their effectiveness decreases. Very recently, some algorithms have been proposed to deal with four or more objectives. In this work, NSGA-III, and MOEA/D [10, 11], which are Many-Objective evolutionary algorithms, have been applied and investigated for optimal tag SNPs selection. The block-based approach is used with different initialization methods e.g. (random, and greedy). The results are compared with some multi-objective algorithms like NSGA-II, and SPEA2. The results of NSGA-III, and MOEA/D are promising for the tag SNP selection problem.

### 1.1 Research contributions

This study has following research contributions:

- Application of many-objective algorithms (NSGA-III and MOEA/D) and demonstrate the significance of using many-objective algorithms for tag SNP problem.
- Evaluate results of many-objective algorithms on benchmarks datasets.
- Comparison of results of many-objective algorithms (NSGA-III and MOEA/D) with previously applied multi-objective algorithms (NSGA-II and SPEA2).
- Demonstrate the significance of using the greedy initialization method for the first time with NSGA-III and MOEA/D algorithms.

The rest of this paper is organized as follows. Section 2 presents the related work for block-based tag SNPs selection. Section 3 formulates the tag SNPs selection as a many-objective problem. Section 4 presents the proposed methodology. Section 5 summarizes the experimental results and presents a discussion on results. Finally, the conclusion and future work are presented in Section 6.

## 2 Related work

This section presents applications of tag SNP selection and previous research on solving tag SNPs selection problem using evolutionary and genetic algorithms.

Tag SNP selection has many applications. Campos et al. [12] identified genomic regions associated with growth traits in Hereford and Braford cattle and for this purpose they used different tag SNP selection techniques. Saher et al. [13] identified SNPs to estimate different genetic parameters such as diversity, pairwise population differentiation, linkage disequilibrium (LD) distribution, and for genome-wide association study for milk yield and body weight traits in the Nili-Ravi dairy bulls. Cattle resistance to ticks is known to be under genetic control with a complex biological mechanism within and among breeds. Bruna et al. [14] identified genomic SNPs associated with tick resistance in Hereford and Braford cattle. The genotype of a single SNP, rs12913832, is the primary predictor of blue and brown eye colors [14]. Olivia et al. [15] investigated variants that explain brown eye color formation in individuals with the rs12913832:GG genotype. Jing et al. [16] investigated the relation between PARP1 haplotypes and lung cancer. They used SNP imputation in their research. Jeong et al. [17] used tag SNPs selection for finding useful information related to the breeding of wild-species tomatoes.

In past, many algorithms and techniques have been proposed for tag SNPs selection problems. Mahdevar et al. [18] proposed a heuristic method based on a genetic algorithm for tag SNPs of haplotype data blocks. The authors used a binary vector of length n which represents a candidate solution in the population of genetic algorithm. The fitness function used was based on a smaller number of haplotype SNPs and it was composed of the Shannon entropy function. Genetic operators such as selection, crossover, and mutation were applied to generate new individuals, then the best candidate solutions that survive to the next iteration were selected.

Gumus et al. [8] proposed a multi-objective tag SNPs selection solution using Pareto Optimality. The proposed method uses Pareto Optimality to solve a multi-objective optimization problem for tag SNPs selection, taking into account classification accuracy and correlation between genomic and geographical distances. Experiments of the proposed method used the SNP dataset from the Human Genome Diversity Project.

Chen et al. [19] proposed a novel method for informative SNPs selection using a genetic algorithm. The proposed method has two phases; first for selecting informative SNPs and second for haplotype reconstruction. First, the authors removed the repeated information of SNPs using linkage disequilibrium values which results in small redundancy, then they used a genetic algorithm for optimization and reconstruction of haplotypes. Authors make use of backpropagation neural networks to rebuild the tagged SNPs, to measure the prediction accuracy of tag SNPs. Liu et al. [20] proposed selection for informative SNPs from large-scale datasets using an improved evolutionary algorithm. The proposed method developed a framework that has two stages to ensure efficiency in selecting informative SNP from a large dataset. In the first stage, the authors developed an improved evolutionary algorithm that selects the informative SNP according to linkage disequilibrium. To reduce the time complexity this stage is independent of the prediction of non-informative SNP. In the second stage, the selection of informative SNPs is done by a forward selection scheme which is based on the performance of prediction. The proposed method has been applied on different large datasets, and experimental results show this method can deal with large datasets effectively. Table 1 shows the summary of the most relevant related work. For each paper, we summarize the methodology, evaluation measures, parameters, and the datasets.

**Table 1:** Summary of the most relevant related work.

Paper	Methodology	Evaluation Measures	Parameters	Dataset
Multi-objective tag SNPs selection using evolutionary algorithms [9]	NSGA-II and MSOPS	Relation between objectives, Range, SumMin, MiniSum, Max and Avg tolerance rate and hamming distance	Population size: 200, Generations: 500	Haplotype block of 1032 SNPs
Tag SNP selection via a genetic algorithm [18]	Genetic algorithm	Overhead 2-8%	Population size: 100, Generations: 100	Chromosome 21 from European population with 34,103 SNPs
Tag SNP Selection Using Similarity Associations Between SNPs [21]	Clonal selection algorithm	Prediction accuracy 96.7%	Generations: 20, pool size: 20, cloning factor: 1	Seven different datasets of different sizes and SNP numbers
Imputation-Aware Tag SNP Selection to Improve Power for Large-Scale, Multi ethnic Association Studies [23]	Genotype-imputation strategies	Imputation accuracy 0.5–7.1%	Thresholds of r2 > 0.5, Minor allele frequency 1%	26 populations from Phase 3 of the 1000 Genomes Project

Ilhan et al. [21] proposed Clonal Selection Algorithm (CLONALG) for tag SNPs selection and compared the results with Particle Swarm Algorithm [22]. Genevieve et al. [23] proposed a novel method for tag SNP selection using genotype-imputation strategies. Shudong et al. [24] constructed an iterative method of selecting tag SNPs based on linkage disequilibrium (TSOILD). They concluded that TSOILD is better than Random Sampling, Greedy Algorithm, and TSMI methods for Tag SNP selection. Ting et al. [9] proposed a multi-objective tag SNPs selection, in which they formulated the tag SNPs problem as multi-objective problem with four objectives. They applied two evolutionary algorithms named non-dominated sorting genetic algorithm-II (NSGA-II) and multiple-single-objective pareto sampling (MSOPS). The data used was haplotype blocks that contain more than 1000 SNPs and haplotype patterns. Authors used greedy initialization to get diverse initial solutions. Results show that the greedy initialization improved the results of multi-objective algorithms.

For two-three objectives, multi-objective algorithms are reasonable enough but as the number of objectives increases to four or more, their effectiveness decreases. Ting el al [9] applied two multi-objective algorithms (NSGA-II and MSOPS) to tag SNP selection problem. However, there are some shortcomings of these two algorithms. The performance of NSGA-II deteriorates when the number of objectives increases. Due to the loss of selection pressure, the convergence of the algorithm is affected. Similarly, slow convergence and lack of diversity of solutions are the two main shortcomings of MSOPS [25]. To address these shortcomings, we suggest NSGA-III and MOEA/D [10, 11], which are many-objective evolutionary algorithms to solve tag SNPs selection problem effectively. In NSGA-III and MOEA/D, these issues are handled using a diverse set of weight vectors, which helps to keep the diverse solutions and maintains an appropriate selection pressure for solving many-objective optimization problems effectively.

## 3 Problem formulation

This section formulates the many-objective tag SNPs selection problem. The number of tag SNPs is just a subset of a large number of SNPs that can distinguish any given haplotype pattern. SNP is the variation at the base position of the genome, and the two possible nucleotide variations are the alleles of that base position [4]. A haplotype block is a set of closely linked alleles/markers on a chromosome that, over evolutionary time, tend to be inherited together [4, 5]. A set of tag SNPs is defined as a set of SNPs that can distinguish any two allele classes [9]. Tag SNPs can be defined as follows.

(Tag SNPs): Given a set of N SNPs *S* = {*S*_1_, …, *S*_*n*_} and M allele classes *P* = {*P*_1_, …, *P*_*m*_}, let *P*_(i,k)_ ∈ {0, 1} denote the *k*^th^ value of haplotype class *P*_*i*_ ∈ {0, 1}^*n*^, where 0 and 1 indicates two possible alleles. A set of tag SNPs *S*^*T*^ is a subset of *S* that can distinguish any two allele classes. That is, for any two allele classes *P*_*i*_ and *P*_*j*_, there exists at least one tag SNP *S*_*k*_ ∈ *S*^*T*^ such that *P*(i,k) ≠ *P*(j,k) [9]. For example

$$\begin{array}{cccccc} & S_{1} & S_{2} & S_{3} & S_{4} & S_{5}\\ P_{1} & 0 & 0 & 1 & 0 & 1\\ P_{2} & 0 & 1 & 1 & 0 & 0\\ P_{3} & 1 & 0 & 0 & 0 & 0\\ P_{4} & 1 & 1 & 0 & 1 & 1 \end{array}$$

is a haplotype block with four different allele classes each with five SNPs. Two SNPs *S*_1_ and *S*_2_ are sufficient to distinguish any of the four allele classes, because *P*_(i,3)_ = *P*_(i,1)_, *P*_(i,4)_ = *P*_(i,1)_ × *P*_(i,2)_, and *P*_(i,5)_ = *P*_(i,1)_ ⊕ *P*_(i,2)_ for i = 1,2,3,4. The tag SNPs selection problem is formally defined as follows: Given M allele classes *P* = {*P*_1_, …, *P*_*m*_} find the smallest tag SNPs set *S*^*T*^ = {*S*_1_, …, *S*_*k*_} and vector function *V*: {0, 1}^*k*^ → {0, 1}^*n*^ such that *V*(*P*_(i,1)_, *P*_(i,2)_, …, *P*_(i,k)_) = *P*_*i*_ for each *P*_*i*_ ∈ *P*.

The multi-objective tag SNPs selection problem is to select a set of tag SNPs that minimizes the total amount of selected SNPs, maximizes their robustness against missing data, maximizes the pair-wise distance among allele classes, and minimizes the variance of these pairwise distances [9]. These four objectives are formally defined below.

### 3.1 Compactness

The first objective is to reduce the number of tag SNPs as much as possible so that the computation time for genotyping platforms is saved [9]. This objective can be defined as

$$\begin{array}{c} min∥S^{T}∥ \end{array}$$ (1)

where ∥*S*^*T*^∥ denotes cardinality of set *S*^*T*^ of selected tag SNPs.

### 3.2 Tolerance

The second objective is to maximize the tolerance for missing data of selected tag SNPs. Let *D*_ij_(*S*^*T*^) denote the number of selected tag SNPs in *S*^*T*^ which are able to distinguish two haplotype patterns *P*_*i*_ and *P*_*j*_. The minimum cardinality of *D*_ij_(*S*^*T*^) among all pairs of alleles gives the number, i.e. *min*(||*D*_ij_(*S*^*T*^)||) − 1, of missing SNPs that the set *S*^*T*^ of tag SNPs can tolerate [26]. The second objective can be defined as

$$\begin{array}{c} max(min∥D_{\text{ij}}\left(S^{T}\right)∥) \end{array}$$ (2)

### 3.3 Dissimilarity

This objective aims to generate dissimilar haplotype backgrounds for distinct allele classes. Similarity on haplotype patterns is calculated by Hamming distance [27] on selected tag SNPs. Let *K*^*T*^ be the index set of *S*^*T*^.

$$\begin{array}{c} S^{T}=\overset{}{\underset{k\in K^{T}}{\cup }}S_{k} \end{array}$$ (3)

The Hamming distance for given two allele classes *P*_*i*_ and *P*_*j*_ is defined by

$$\begin{array}{c} H\left(P_{i},P_{j}\right)=\sum_{k\in K^{T}}^{}\left|P_{i,k}-P_{j,k}\right| \end{array}$$ (4)

This objective tries to maximize average Hamming distance for all pairs of haplotype patterns.

$$max\bar{H}$$

The average hamming distance can be defined as

$$\begin{array}{c} \bar{H}=\frac{1}{\left(\begin{array}{c} m\\ 2 \end{array}\right)}\sum_{0\leq i<j\leq m}^{}H\left(P_{i},P_{j}\right) \end{array}$$ (5)

### 3.4 Balance

This objective aims to balance tag SNPs for every haplotype pattern. It minimizes a variance on the distances calculated on the third objective, between all pairs of haplotype patterns for a given solution of tag SNPs [9]. This objective will produce unbiased tag SNPs which can be distinguishing patterns. So, the fourth objective is minimizing the variance by

minVar(H)

with

$$\begin{array}{c} var\left(H\right)=\frac{1}{\left(\begin{array}{c} m\\ 2 \end{array}\right)}\sum_{0\leq i<j\leq m}^{}{\left(H\left(P_{i},P_{j}\right)-\bar{H}\right)}^{2} \end{array}$$ (6)

## 4 Proposed methodology

For solving the tag SNPs with many objectives, the proposed method uses different initialization methods and integrates them into evolutionary many-objective optimization algorithms NSGA-III and MOEA/D [10, 11].

Evolutionary algorithms (EA) use the techniques and methods inspired by biological evolution, like selection, reproduction, mutation, and recombination. Fitness function determines the goodness of solutions. EA repeatedly applies its operators in each iteration, which improves the candidate solutions in each iteration and moves the solutions toward the optimal solution.

### 4.1 Similarities and differences between the existing and proposed methods

Ting et al. [9] considered four objectives in the tag SNPs selection problem, and solved the optimization problem using NSGA-II, and MSOPS, which are two famous evolutionary algorithms. NSGA-II is a multi-objective Pareto-based approach that has been successfully applied to solve optimization problems with two or three objectives. However, as the number of objectives increases to four or more, it gets hard to differentiate between different solutions in the population and a large fraction of solutions become non-dominated trade-off solutions. Due to the loss of the selection pressure, NSGA-II like other multi-objective algorithms is unable to differentiate between solutions, which slows down the search and overall degrades the performance of the algorithm. The MSOPS, an aggregation-based approach exhibiting the advantages of both aggregation and Pareto-based evolutionary algorithms, is used to solve many-objective optimization problems. Nevertheless, slow convergence and lack of diversity of solutions are the two main shortcomings of MSOPS [25].

To address the aforementioned problems of NSGA-II, and MSOPS, we suggest two widely used many-objective evolutionary algorithms NSGA-III [10], and MOEA/D [11]. Similar to NSGA-II and MSOPS, NSGA-III and MOEA/D are Pareto-based, and aggregation-based search methods, respectively. NSGA-III is an extension of NSGA-II to solve many-objective optimization problems (with 4 or more objectives) effectively. The NSGA-III algorithm [10] can be used for many-objective problems with at most 15 objectives. Similar to NSGA-II, NSGA-III uses fast non-dominated sorting to rank solutions on fronts. To select a subset of solutions from the same front, crowding distance is used in NSGA-II. On the other hand, NSGA-III replaces the crowding distance procedure with an improved method that uses uniformly spread reference vectors and niching to maintain a balanced search in all directions of the search space. The NSGA-II generates near-optimal solutions for optimization problems with two or three objectives. But the performance of NSGA-II is not effective for a large number of objectives. The reason is the augmented likelihood of non-dominance. In NSGA-III, this issue is dealt with using a diverse set of weight vectors, which helps to keep the diverse solutions and maintains an appropriate selection pressure for solving many-objective optimization problems effectively. MSOPS and MOEA/D both use an aggregation-based approach for the fitness assignment; however, uniformly spread weight vectors are used in MOEA/D, which help to explore different regions in the search space and maintain a diverse set of solutions. MOEA/D decomposes a multi-objective optimization problem into many single-objective optimization subproblems, then it optimizes these subproblems simultaneously. Each solution is associated with a subproblem, and each subproblem is optimized by using information from its neighborhoods. In MOEA/D, a set of weight vectors is used to specify several subregions in the objective space. Furthermore, each weight vector also defines a subproblem for fitness evaluation. The parent population is updated in a steady-state scheme, where only one offspring solution is considered each time.

The components and operators of the proposed many-objective GAs are described in the following subsections.

### 4.2 Representation and fitness function

One candidate solution which has a set of selected tag SNPs, is a binary vector or chromosome *c* = [*c*_1_, …, *c*_*n*_], where single gene *c*_*k*_ ∈ {0, 1} denotes if SNP *S*_*k*_ is selected or not. If it is selected then *c*_*k*_ is 1 else it is 0. As a result, every binary vector of length n is a feasible solution, and evolutionary operators must be able to produce it. For example for the candidate *c* = [ 1 0 1 0 0 ], with *n* = 5, the number of tag SNPs *S*^*T*^ = {*S*_1_, *S*_3_} is 2 and only the first, and third SNPs are selected.

The many-objective tag SNPs selection problem considers four objectives (compactness, tolerance, dissimilarity, and balance). Having many objectives, the proposed method uses the concept of dominance. Given two chromosomes (a, and b), chromosome (a) is better or dominates chromosome (b), if one more objective of (a) are better than objectives of (b) and (a) is not worse compared to chromosome (b) on any of the rest of the objectives. So a superior rank can be assigned to chromosome (a). In case neither chromosome (a) nor chromosome (b) is dominated, both chromosomes are nondominated and both will be at the same rank. The set of nondominated solutions is known as the Pareto front. The fitness function of a solution (a) for M objectives can be calculated as follows:

$$\begin{array}{c} f(a)=\sum_{i=1}^{M}rank\left(f_{i}\left(a\right)\right) \end{array}$$ (7)

where *rank*(*f*_*i*_(*a*)) provides the rank of (a) in a set of solutions according to objective *f*_*i*_. The best objective value is ranked 1 and the worst objective value is ranked N. Given two solutions (a, and b), if *f*(*a*)<*f*(*b*), then (a) dominates (b) [28].

### 4.3 Initialization

The first step in Genetic Algorithms (GAs) is to initialize a set of chromosomes as the initial population. The initialization usually generates chromosomes randomly. Conventional multi-objective GAs result in candidate solutions being gathered close to the middle of the objective space. An initialization based on the greedy method [9] addresses the issue of random initialization. The two different initialization methods (random, greedy) have been investigated in this study.

The greedy initialization defines the ability to distinguish of an SNP *S*_*k*_ as the number of allele pairs it can distinguish. For example,

$$\begin{array}{ccccccccc} & S_{1} & S_{2} & S_{3} & S_{4} & S_{5} & S_{6} & S_{7} & S_{8}\\ P_{1} & 0 & 0 & 1 & 0 & 1 & 0 & 0 & 1\\ P_{2} & 0 & 1 & 1 & 0 & 0 & 0 & 1 & 1\\ P_{3} & 1 & 0 & 0 & 0 & 0 & 1 & 0 & 0\\ P_{4} & 1 & 1 & 0 & 1 & 1 & 0 & 1 & 1\\ P_{5} & 0 & 0 & 1 & 1 & 1 & 0 & 0 & 0\\ P_{6} & 1 & 0 & 1 & 0 & 0 & 0 & 1 & 1\\ distinguishability & 9 & 8 & 8 & 8 & 9 & 5 & 9 & 8 \end{array}$$

if the ability to distinguish SNP *S*_1_ is 9 then it means that the SNP *S*_1_ can distinguish 9 different allele pairs, while SNP *S*_6_ can distinguish 5 different allele pairs.

Greedy initialization works as follows. A variant of the set cover problem can be used to solve the tag SNPs selection problem [6]. In the set cover problem, each element must be covered by a specified number of sets, it is known as coverage. SNPs are sorted based on distinguishability in descending order. For each chromosome, sorted SNPs are iteratively selected until all elements are covered. In the end, the initial population will have chromosomes with respect to different coverage. So, this greedy initialization would result in a good initial population with broader solutions and promising exploration of solution space than random initialization.

### 4.4 Genetic operators

Genetic Algorithm (GA) selects chromosomes of the initial population as parents, then performs crossover and mutation operations to generate the offspring. The binary tournament is used for selection, it picks the best chromosome to be a parent from two random chromosomes. Two parents are selected in this manner, then GA uses both crossover and mutation operators on selected parents to generate two new offspring. Crossover combines parts of the parents, whereas mutation changes the offspring a little bit. In this work, uniform crossover [29] is used. The bit-flip mutation flips genes (*i*.*e*.0 → 1, 1 → 0) given mutation rate *P*_*m*_. GA generates a set of offspring, combines parents and offspring, then selects the fittest chromosomes for the next generation. After generating a set of offspring, GA applies the principle of ‘survival of the fittest. Restated, only the fittest chromosomes are selected to survive into the next generation. This article makes use of the above-stated strategies of NSGA-III and MOEA/D for survival selection concerning many objectives.

## 5 Experimental results and discussion

This work leads a progression of analyses to assess the proposed techniques on the many-objective tag SNPs selection problem. All experiments use the data of population haplotypes from Hinds et al. (2005) dataset [2], where they characterized whole-genome patterns of common human DNA variation by genotyping 1,586,383 SNPs in three population samples, Americans of European, African, and Asian ancestry. Haplotype patterns are combined together to form one haplotype block. This proposed method uses those haplotype blocks whose number of SNPs is greater than 1000. In these experiments, a block of 1032 SNPs is used.

**Fig 1:**
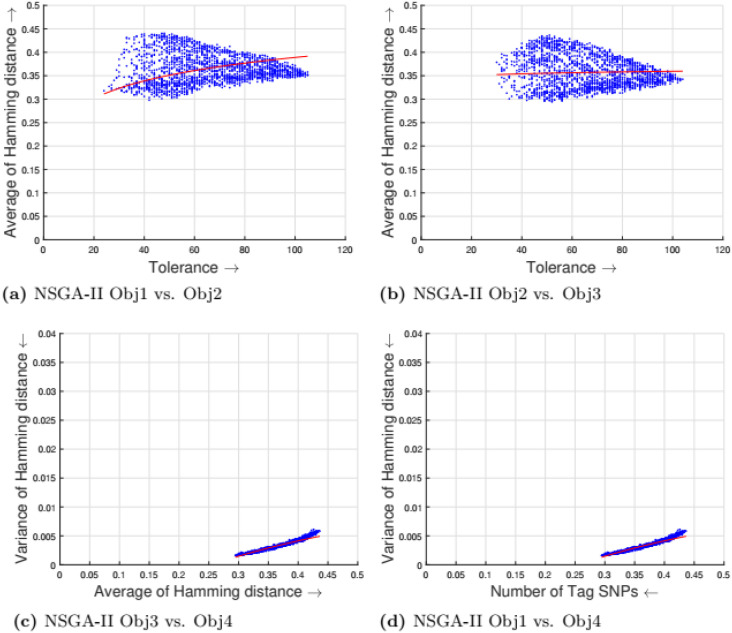
Experimental results of NSGA-II for the tag SNPs selection using random initialization. Obj1 stands for the number of tag SNPs, Obj2 is the tolerance for missing data, Obj3 measures the average Hamming distance between alleles, and Obj4 controls the variance of detection power in each allele. (a) NSGA-II obj1 vs. Obj2, (b) NSGA-II Obj2 vs. Obj3, (c) NSGA-ll Obja vs. obj4, (d) NSCA-II Ohj1 vs. Obj4.

**Fig 2:**
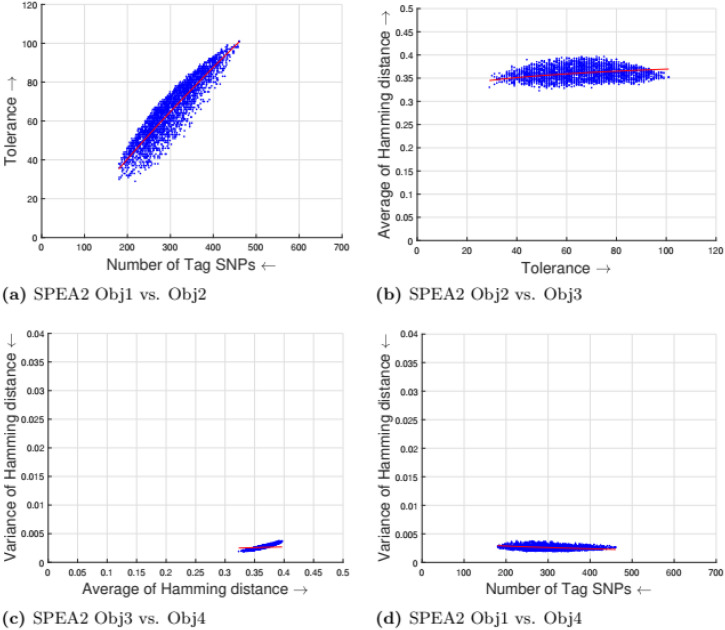
Experimental results of SPEA2 for the tag SNPs selection using random initialization. Obj1 stands for the number of tag SNPs, Obj2 is the tolerance for missing data, Obj3 measures the average Hamming distance between alleles, and Obj4 controls the variance of detection power in each allele. (a) SPEA2 obj1 vs. Obj2, (b) SPEA2 Obj2 vs. Obj3, (c) SPEA2 Obj3 vs. Obj4, (d) SPEA2 Obj1 vs. Obj4.

**Fig 3:**
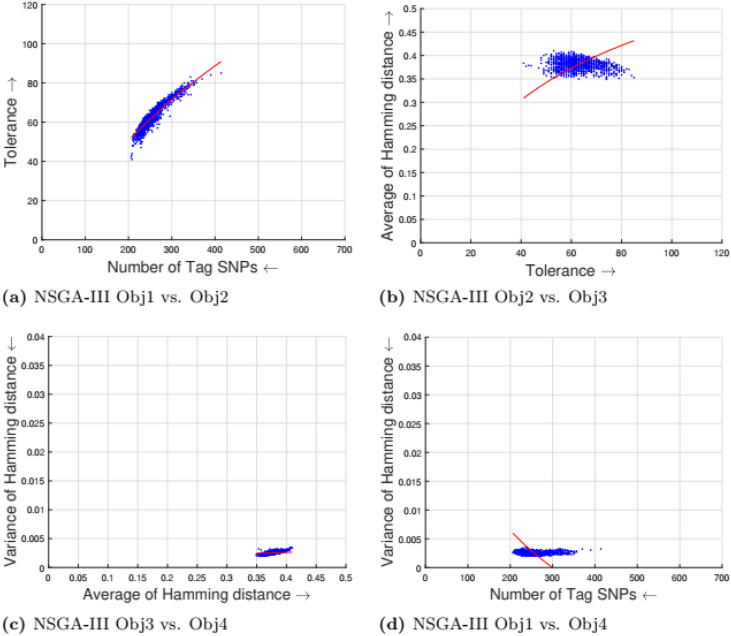
Experimental results of NSGA-III for the tag SNPs selection using random initialization. Obj1 stands for the number of tag SNPs, Obj2 is the tolerance for missing data, Obj3 measures the average Hamming distance between alleles, and Obj4 controls the variance of detection power in each allele. (a) NSGA-III Obj1 vs. Obj2, (b) NSGA-III Obj2 vs. Obj3, (c) NSGA-III Obj3 vs. Obj4, (d) NSGA-III Obj1 vs. Obj4.

**Fig 4:**
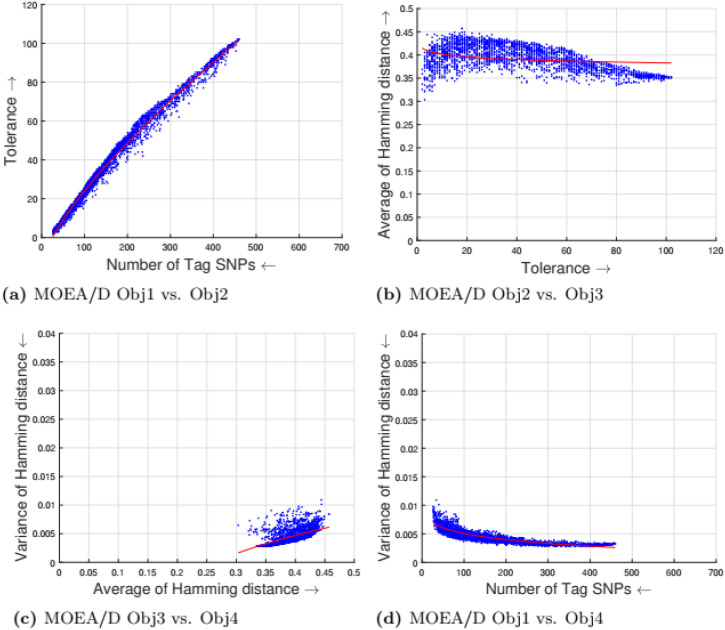
Experimental results of MOEA/D for the tag SNPs selection using random initialization. Obj1 stands for the number of tag SNPs, Obj2 is the tolerance for missing data, Obj3 measures the average Hamming distance between alleles, and Obj4 controls the variance of detection power in each allele. (a) MOEA/D Obj1 vs. Obj2, (b) MOEA/D Obj2 vs. Obj3, (c) MOEA/D Obj3 vs. Obj4, (d) MOEA/D Obj1 vs. Obj4.

**Fig 5:**
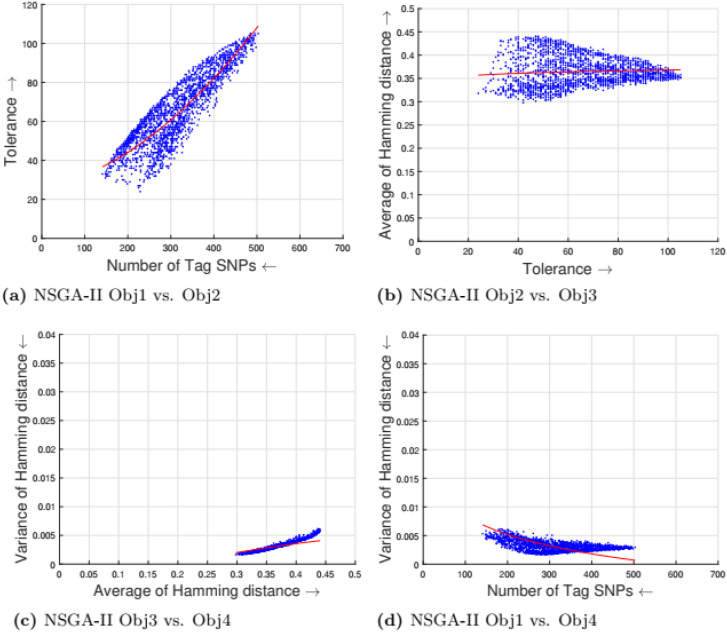
Experimental results of NSGA-II using greedy initialization for the tag SNPs selection problem. Obj1 stands for the number of tag SNPs, Obj2 is the tolerance for missing data, Obj3 measures the average Hamming distance between alleles, and Obj4 controls the variance of detection power in each allele. (a) NSGA-II Obj1 vs. 0bj2, (b) NSGA-II Obj2 vs. 0bj3, (c) NSGA-II Obj3 vs. 0bj4, (d) NSGA-II Obj1 vs. 0bj4.

**Fig 6:**
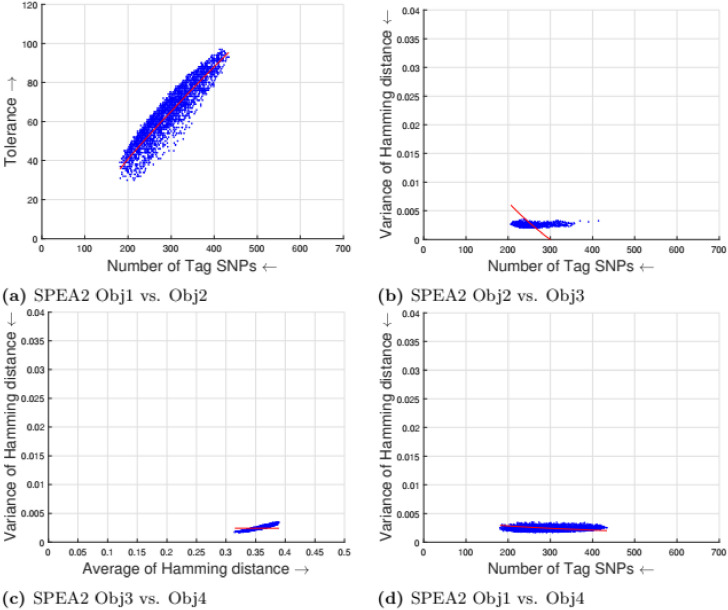
Experimental results of SPEA2 using greedy initialization for the tag SNPs selection problem. Obj1 stands for the number of tag SNPs, Obj2 is the tolerance for missing data, Obj3 measures the average Hamming distance between alleles, and Obj4 controls the variance of detection power in each allele. (a) SPEA2 Obj1 vs. obj2, (b) SPEA2 Obj2 vs. obj3, (c) SPEA2 Obj3 vs. obj4, (d) SPEA2 Obj1 vs. obj4.

**Fig 7:**
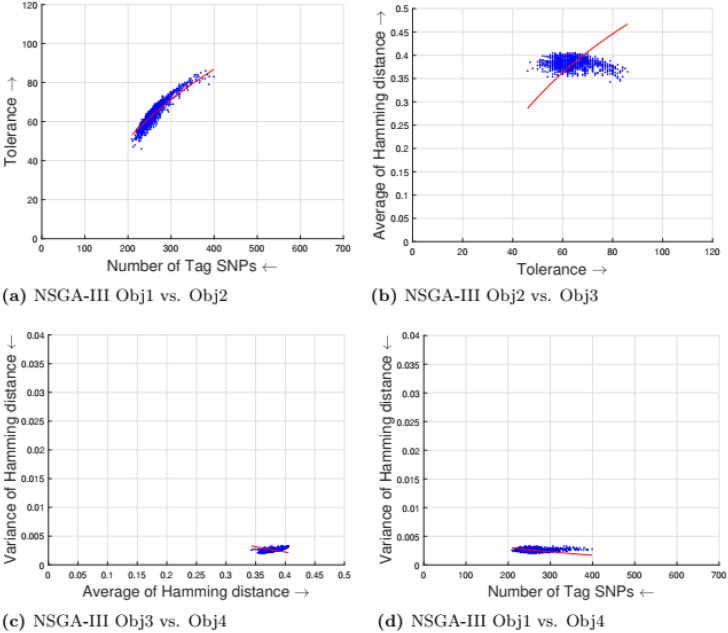
Experimental results of NSGA-III using greedy initialization for the tag SNPs selection problem. Obj1 stands for the number of tag SNPs, Obj2 is the tolerance for missing data, Obj3 measures the average Hamming distance between alleles, and Obj4 controls the variance of detection power in each allele. (a) NSGA-Ill Obj1 vs. Obj2, (b) NSGA-Ill Obj2 vs. Obj3, (c) NSGA-Ill Obj3 vs. Obj4, (b) NSGA-Ill Obj1 vs. Obj4.

**Fig 8:**
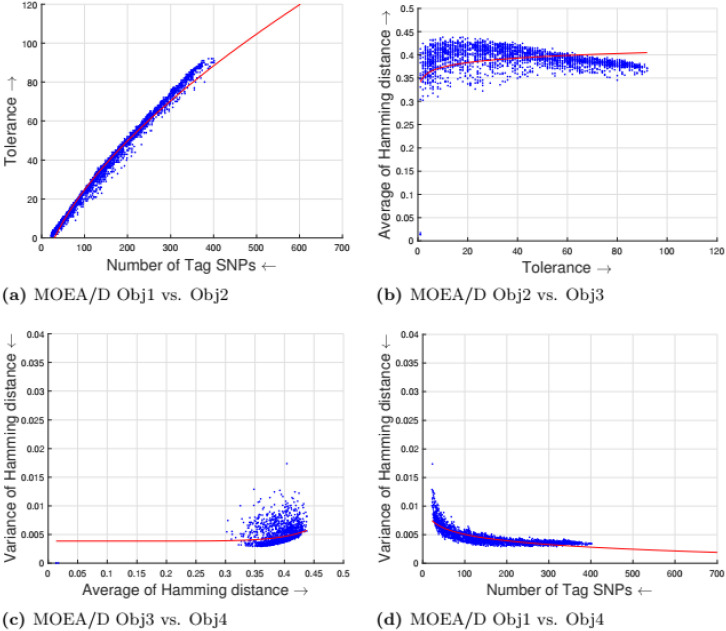
Experimental results of MOEA/D using greedy initialization for the tag SNPs selection problem. Obj1 stands for the number of tag SNPs, Obj2 is the tolerance for missing data, Obj3 measures the average Hamming distance between alleles, and Obj4 controls the variance of detection power in each allele. (a) MOEA/D Obj1 vs. Obj2, (b) MOEA/D Obj2 vs. Obj3, (c) MOEA/D Obj3 vs. Obj4, (b) MOEA/D Obj1 vs. Obj4.

Table 2 shows the parameter setting for the proposed many-objective evolutionary algorithms based on NSGA-II, NSGA-III, SPEA2, and MOEA/D. All the compared optimization algorithms are set as suggested by the authors in the the study [9]. Experimental results for four objectives are shown in Figs 1 to 8. Each plot shows the relation between two objectives, where each axis represents one objective and arrows show the direction of optimization for a given objective. Moreover, a regression line of the power function *y* = *ax*^*b*^ + *c* is plotted to show the spread and diversity of solutions.

**Table 2:** Parameter setting for experiments of many-objective evolutionary algorithms. These parameters are set according to the study [9].

Parameter	Value
Haplotype size	1032 SNPs
Representation	Binary string
Population size	200
Offspring size	200
Neighborhood size	15
Crossover	*p*_*c*_ = 0.7
Mutation	*p*_*m*_ = 1/*l*, l is length of vector
Termination	500 generations
Initialization	Random/greedy
Number of algorithms	4
Number of runs	5
Total Number of experiments	40

Figs 1 to 4 illustrate the experimental results for random initialization on NSGA-II, NSGA-III, SPEA2, and MOEA/D. Solutions obtained by NSGA-II, NSGA-III, and SPEA2 are close to the center of the objective space, whereas the solutions obtained by MOEA/D are more spread across the objective space. Specifically, algorithms NSGA-II, NSGA-III, and SPEA2 fail to find solutions with fewer than 150-170 SNPs, but MOEA/D can find solutions with fewer (almost 25) tag SNPs. The three algorithms do not cover objective space like MOEA/D. To resolve this issue, the distribution of initial solutions is required to cover more diversity from objective space.

Figs 5 to 8 illustrate the experimental results for greedy initialization on NSGA-II, NSGA-III, SPEA2, and MOEA/D. The solutions obtained by greedy initialization show more diversity which is important for many objective optimization problems. The diversity gives many options to users for choosing a suitable solution for different scenarios. Greedy initialization gives SNPs with high distinguishability and increases the dissimilarity between haplotype patterns (the third objective) which leads toward optimal solutions.

When the initial population is randomly initialized, the individuals (solution vectors) are uniformly spread throughout the search space. The main difference in performance is due to the difference in parent selection procedures of NSGA-II and MOEA/D. Each new solution (offspring) is generated using the selected parents. In random initialization, as the solutions are far away from each other, there is a high probability that the selected parent solutions are distant from each other. If the parents are distant from each other, the generated offspring will most likely be quite different from their parents and they may lie in the far regions of the search space that may enhance exploration thus it can take more generations to converge to the optimal. On the contrary, in MOEA/D, while updating a solution (offspring generation), the parent solutions are selected from the neighborhood of that solution. The generated solution will not be very different and it exploits the information from both parents and the convergence to the near-optimal is fast as compared to the NSGA-II. In the case of greedy initialization, NSGA-II may get more advantage because the selected parents may be already close to the local optima, and the generated offspring can converge in less number of iterations as compared to the random initialization. On the other hand, as MOEA/D already selects parent solutions from the neighborhood of a solution, greedy initialization does not help much in the exploitation of the search space.

Table 3 shows the overall coverage (range and standard deviation) for 4 objectives of NSGA-II, SPEA2, NSGA-III, and MOEAD using both random and greedy initialization. The above outcomes show the advantages of greedy initialization over random initialization using NSGA-II, NSGA-III, SPEA2, and MOEA/D algorithms for solving the tag SNPs selection problem as a many-objective optimization problem.

**Table 3:** Spread for objectives 1 to 4 of NSGA-II, SPEA2, NSGA-III, and MOEAD using RI random initialization in Fig 1 and GI greedy initialization in Fig 2.

Algo.		Obj1		Obj2		Obj3		Obj4	
		Range	Std.	Range	Std.	Range	Std.	Range	Std.
NSGA-II	RI	319	82	74	16	0.1419	**0.0338**	0.0045	0.0010
	GI	362	81	81	18	0.1427	0.0310	0.0045	0.0009
SPEA2	RI	281	59	72	15	0.0737	0.0135	0.0019	0.0003
	GI	253	58	67	15	0.0749	0.0153	0.0018	0.0004
NSGA-III	RI	208	29	44	6	0.0606	0.0114	0.0015	0.0003
	GI	190	29	40	6	0.0628	0.0112	0.0013	0.0003
MOEAD	RI	434	**113**	**100**	**26**	0.1541	0.0263	0.0081	0.0013
	GI	**723**	101	91	25	**0.4244**	0.0242	**0.0174**	**0.0015**

### 5.1 Relation between objectives

This section is about the findings of four objectives and their trade-off from the experimental results.

#### 5.1.1 Compactness and tolerance

The first objective (compactness) and second objective (tolerance) have a trade-off as having a high tolerance for missing data requires more SNPs to be selected. The trade-off is linear between compactness and tolerance (see Figs 5(a), 6(a), 7(a) and 8(a)). Furthermore, in genotyping platforms the tolerance for missing SNPs is limited. Therefore, taking into account the limit of tolerance for different genotyping platforms, the proposed method can increase the number of selected tag SNPs with the lowest cost. But, when the tolerance limits are high a large number of tag SNPs is needed.

#### 5.1.2 Tolerance and dissimilarity

There is a trade-off between the second objective (tolerance) and the third objective (dissimilarity) as shown in experimental results (see Figs 5(b), 6(b), 7(b) and 8(b)). The reason is that high tolerance for missing data requires more tag SNPs to be selected, but on other hand, having more tag SNPs does not necessarily increase the hamming distance. The experimental results show solutions considering these two objectives to be quite diverse, and no relationship can be built.

#### 5.1.3 Dissimilarity and balance

There is a trade-off between the third objective (dissimilarity) and the fourth objective (balance) as shown in experimental results (see Figs 5(c), 6(c), 7(c) and 8(c)). Having a small average distance between haplotype patterns gives a low variance, and when the average distance increases, the detection power increases as well. The variance is exponentially increased when the average distance increases.

#### 5.1.4 Compactness and balance

There is a trade-off between the first objective (compactness) and the fourth objective (balance or variance of hamming distance). Both objectives are minimizing objectives, but to increase the detection power of each haplotype pattern by minimizing the variance of hamming distance, more tag SNPs need to be selected. The trade-off in different experimental results (see Figs 5(d), 6(d), 7(d) and 8(d)) show that when the number of selected tag SNPs is increased, a high minimization in the variance of hamming distance is achieved. When there are sufficient tag SNPs, then the change in balance will be less important. Hence, a slight increase in the number of selected tag SNPs is enough to have a good balance in each haplotype pattern.

### 5.2 Effectiveness of proposed greedy initialization method

This work uses the performance measures proposed by [30, 31]. Both random and greedy initialization methods are used to compare the performance of the proposed method which uses NSGA-II, NSGA-III, SPEA2, and MOEA/D algorithms for tag SNPs selection problem using many objectives. Before applying the evaluation measures, the second and fourth objectives should be converted into minimization objectives. All objectives are normalized and all four algorithms using two different initialization were executed five times. The evaluation measures used for comparing results for different algorithms are Hypervolume, Range, SumMin, MinSum, Tolerance rate, and Average Hamming distance. Each of these evaluation measures is described in the following sections.

#### 5.2.1 Range

**Fig 9:**
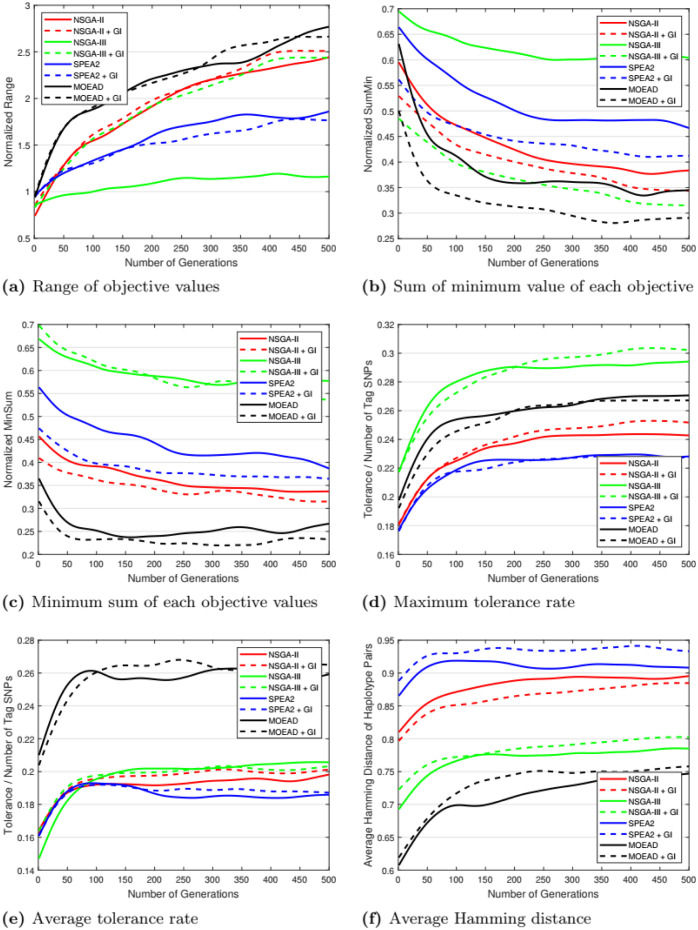
Performance comparison of algorithms. GI stands for greedy initialization. (a) Range of objective values, (b) Sum of minimum value of each objective, (c) Minimum sum of each objective values, (d) Maximum tolerance rate, (e) Average tolerance rate, (f) Average Hamming distance.

The range of each objective is the difference between the highest and the lowest values of an objective. The range of objective values is summed as one range in each generation. The range is important to measure the diversity of solutions in the objective space in each generation. Fig 9(a) and Table 4 show experimental results for range, considering NSGA-III, there is an improvement in the range with greedy initialization over the random initialization. However, the performance of SPEA2, MOEA/D, and NSGA-II is almost the same for both cases. As far as the results of NSGA-III are concerned, the greedy initialization improves the spread of solution range.

**Table 4:** Performance comparison of algorithms using measures of range, SumMin, and MinSum. GI stands for greedy initialization, RI stands for random initialization.

Algo	Initialization	Range		SumMin		MinSum	
		Range	Std.	Range	Std.	Range	Std.
NSGA-II	RI	1.7877	0.4359	0.2331	0.0542	0.1310	0.0296
	GI	1.8090	**0.4522**	0.2005	0.0483	0.1114	0.0229
SPEA-2	RI	1.0429	0.2499	0.2135	0.0506	0.1896	**0.0404**
	GI	1.0608	0.2128	0.1768	0.0354	0.1311	0.0252
NSGA-III	RI	0.4639	0.0923	0.1304	0.0249	0.1244	0.0243
	GI	1.7563	0.4390	0.1838	0.0443	0.1899	0.0390
MOEAD	RI	**2.1533**	0.3981	**0.4092**	**0.0603**	**0.1968**	0.0238
	GI	2.1382	0.4080	0.3018	0.0435	0.1578	0.0179

#### 5.2.2 SumMin

SumMin sums the minimum values of all objectives on a given iteration, this measure shows the convergence of solutions toward the Pareto front around its marginal region. Fig 9(b) and Table 4 show experimental results for SumMin. Greedy initialization produces solutions with possible minimum objective values. All algorithms have improvement with greedy initialization, specifically MOEA/D with minimum SumMin. When the initial population is randomly initialized, the individuals (solution vectors) are uniformly scattered throughout the search space, and it requires more generations to converge to the optimal.

#### 5.2.3 MinSum

MinSum is calculating the sum of the four objective values of each solution then takes the minimum sum value in a single generation, this measure shows the convergence of solutions toward the Pareto front around its central region. Fig 9(c) and Table 4 show experimental results for MinSum. MOEA/D has the best solutions with a minimum sum, MOEA/D yields much better results than NSGA-II, NSGA-III, and SPEA2. The greedy initialization improves SPEA2 and NSGA-II to exploit solutions as well, but for NSGA-III greedy initialization does not help or give any improvement. Hence, results using greedy initialization are better as compared to random initialization using MinSum measure.

#### 5.2.4 Tolerance rate

Tolerance rate is the result of dividing the second objective (tolerance) by the first objective (selected tag SNPs). Its purpose is to show how the algorithm is capable of finding the tag SNPs set given a high tolerance rate. Fig 9(d) and Table 5 show experimental results for maximum tolerance rate. Regardless of the use of greedy initialization, the NSGA-III algorithm gives the highest tolerance rate as compared to the rest of the algorithms.

**Table 5:** Performance comparison of algorithms using measures of maximum tolerance rate, average tolerance rate, and average hamming distance. GI stands for greedy initialization, RI stands for random initialization.

Algo	Initialization	Max Tolerance Rate		Avg Tolerance Rate		Avg Hamming distance	
		Range	Std.	Range	Std.	Range	Std.
NSGA-II	RI	0.0692	0.0146	0.0485	0.0060	0.1022	0.0191
	GI	0.0781	0.0170	0.0520	0.0075	0.1061	0.0195
SPEA-2	RI	0.0633	0.0113	0.0487	0.0056	0.0773	0.0094
	GI	0.0593	0.0108	0.0474	0.0054	0.0769	0.0098
NSGA-III	RI	0.0859	0.0160	0.0726	0.0120	0.1083	0.0191
	GI	**0.0937**	**0.0204**	0.0525	0.0075	0.0962	0.0177
MOEAD	RI	0.0891	0.0149	0.0816	0.0099	0.1500	0.0309
	GI	0.0895	0.0173	**0.0879**	**0.0130**	**0.1523**	**0.0326**

Fig 9(e) and Table 5 show experimental results for the average tolerance rate. Taking average tolerance rate into consideration, MOEA/D outperforms all the compared algorithms, and NSGA-III performs slightly better than NSGA-II, and SPEA2.

#### 5.2.5 Average hamming distance

This is the measure of the third objective (Average Hamming distance). Using all pairs of haplotype patterns, the average Hamming distance is calculated in each experiment. An increase in the hamming distance of solutions is preferred. Fig 9(f) and Table 5 show experimental results for average hamming distance. SPEA2 and NSGA-II perform better on this measure. Although MOEA/D like other algorithms does not perform well on this measure, it shows good results in other measures like tolerance and balance.

Overall MOEA/D algorithm gives superior results as compared to other algorithms in most cases. NSGA-III outperforms NSGA-II and other compared algorithms on maximum tolerance rate, and SPEA2 outperforms all algorithms on average hamming distance. Moreover, NSGA-III with greedy initialization outperforms NSGA-II and SPEA2 for the SumMin measure. Compared with multi-objective algorithms (NSGA-II, and SPEA2), NSGA-III also shows equal and better performance on Range, and MinSum measures, respectively. Overall many-objective algorithms (MOEA/D and NSGA-III) outperform multi-objective algorithms for optimization problems with four objectives (tag SNP problem).

### 5.3 Genetic parameter change

**Fig 10:**
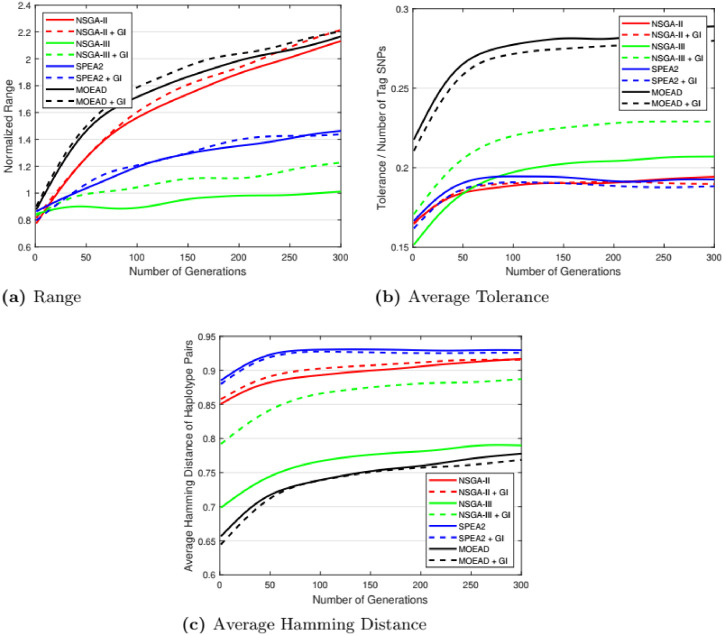
Performance comparison of algorithms. Population size = 100 and Generation = 300. (a) Range, (b) Average Tolerance, (c) Average Hamming Distance.

Besides existing experiments in which the population size and the number of generations are set to 200, and 500 respectively, we have also included experimental results with a population size = 100, and the number of generations = 300. Fig 10 shows the results of different algorithms with new parameters. A comparison of Figs 9 and 10 shows that there is no significant difference in the relative performance of the algorithms.

## 6 Conclusions

Genotyping of single-nucleotide polymorphisms (SNPs) is necessary for finding associations in the genome. These associations help in finding genetic role in many complex diseases. A small subset of SNPs is selected and used but this subset should be selected using some objectives. Tag SNP selection is an NP-hard problem since it has many conflicting objectives. This study proposes to solve the tag SNP prediction problem using many-objective optimization algorithms. Experiments are conducted on benchmark datasets and the results are compared with NSGA-II, and SPEA2, which are highly cited multi-objective evolutionary algorithms. NSGA-II has been already used to solve tag SNP problem in a previous research. Results show the superior performance of our proposed many-objective evolutionary algorithms (MOEA/D and NSGA-III). The experimental results indicate that MOEA/D outperforms NSGA-II, NSGA-III, and SPEA2 in most of the cases. This study also investigates different initialization methods like greedy and random initialization. The outcomes show the advantages of greedy initialization over random initialization using NSGA-III, SPEA2, and MOEA/D to solve the tag SNPs selection as many-objective optimization problem. The results from the proposed method have many non-dominated solutions. It provides users with many options to choose from a set of tag SNPs that are suitable for their needs and different genotyping platforms. One of the possible future directions is that the capability of the proposed method can be investigated in a non-block-based tag SNPs approach.
